# Nicotine addiction as a moral problem: Barriers to e-cigarette use for smoking cessation in two working-class areas in Northern England

**DOI:** 10.1016/j.socscimed.2019.112498

**Published:** 2019-10

**Authors:** Frances Thirlway

**Affiliations:** a University of York, Sociology Department, UK; b Durham University, Anthropology Department, UK

**Keywords:** UK, Smoking cessation, Electronic cigarettes, Addiction, Ethnography, Health inequalities, Thrift as care

## Abstract

Tobacco use in high-income countries correlates with socio-economic disadvantage, but although switching to electronic cigarettes could be a safer alternative, little is known about barriers to use. Drawing on eighteen months of data collection in two areas of Northern England in 2017/18 including ethnography and interviews with 59 smokers and e-cigarette users, I show that concern about continued nicotine addiction either deterred working-class smokers from switching to e-cigarettes or dictated the conditions of their use. Research participants were unhappy about addiction both as loss of control experienced as moral failure and as neglect of financial responsibilities i.e. role performance failure in relation to family responsibilities, or what I call ‘thrift as care’. They reduced the moral burden of addiction by lowering nicotine content, rejecting pleasure and minimising expenditure. They chose the cheapest possible tobacco, switched from combusted tobacco to cheaper e-cigarettes and bought cheap e-cigarettes and liquids. For working-class smokers, minimising spend on what they perceive negatively as addiction may be a greater moral concern than reducing health risk. I conclude that ensuring that vaping is significantly cheaper than smoking may be key to addressing health inequalities linked to tobacco use.

## Highlights

• Working-class smokers avoided e-cigarettes because of concern about addiction.
• Those who did switch preferred a medical to a recreational model of use.
• Users reduced nicotine content, minimised spending and avoided exotic flavours.
• Failure of willpower and role performance (thrift as care) created addiction shame.
• Ensuring that vaping is cheaper than smoking is key to harm reduction.

## 1 Introduction

Whilst smoking rates in high-income countries have declined over the past thirty years, they remain high amongst the poorest (West et al., 2019) and contribute significantly to health inequalities (Kulik et al., 2013). Since smokers with lower socio-economic status (SES) consistently score highly on indices of addiction (Reid et al., 2010), it has been hypothesised that they might benefit disproportionately from electronic cigarettes, which administer nicotine, the addictive ingredient of tobacco, in a safer, non-combusted form (Fairchild et al., 2014). This is an example of the harm reduction approach to addiction according to which public health authorities should reduce harm from addictive substances by promoting safer way of taking them (Marlatt, 1996). Key research questions in relation to e-cigarettes therefore include not only whether they can improve smoking cessation rates at the population level, but crucially whether they can address the fact that smoking rates remain highest amongst the most deprived (Kock et al., 2018; Hartwell et al., 2017; Thirlway, 2016).

The UK is of particular interest because it is probably the country in which public health authorities have most explicitly endorsed e-cigarettes as a smoking cessation tool (Green et al., 2016). E-cigarettes are now the most commonly-used smoking cessation method in the UK (West et al., 2019) and rates of use by SES have been converging (Kock et al., 2018) following greater initial take-up by high-SES smokers (Hartwell et al., 2017). However, a third of UK smokers have not tried e-cigarettes and the fear of swapping one addiction for another is at the top of their list of concerns (ASH, 2018; Rooke et al., 2016; Farrimond, 2017; Sherratt et al., 2015; Lucherini et al., 2017; Thirlway, 2016). Their worries reflect a genuine contradiction: like nicotine replacement therapy (NRT), e-cigarettes are both an addictive product themselves and a cure for addiction (Bell and Keane, 2012 p 244). Unlike NRT, which is designed and marketed as a time-limited smoking cessation tool, the e-cigarette has acquired disruptive status from uncertainty as to whether it is a temporary cessation aid, a long-term harm reduction device or a recreational item (Rooke et al., 2016 p. e62-e63).

Social scientists take the view that health behaviours are symbolic practices which can only be understood within a wider cultural framework (Poland et al., 2006; Crossley, 2001; Goldade et al., 2012; Prentice, 2010). They have long argued that addiction is socially produced (Quintero and Nichter, 1996; Room, 2003; Keane, 2002; Fraser et al., 2014) in contrast to both the disease model of addiction (Heather et al., 2018) and the moral model which views substance use as a bad choice (Berridge, 2013). However, we direct our lives and weigh our daily decisions in terms of moral questions (Holdsworth and Morgan, 2007; Sayer, 2005; Miller, 1998 p. 19) and descriptions of drug cultures and identities which strive to avoid moralism may overlook the extent to which loss of self-control is a moral problem for drug-users *themselves* (Weinberg, 2011 p. 302). This recent critique echoes earlier concerns that fine-grained ethnographic studies can neglect ethical issues, whether by deflating the problems associated with substance use (Room, 2003) or failing to recognise their connection to global social processes (Singer, 1986; Hunt and Barker, 2001). Weinberg argues that only a fraction of studies speaks explicitly to the nature of addiction itself (Weinberg, 2011 p. 302), and for this I turn to perspectives from philosophy.

Whilst much of the philosophy of addiction is concerned with free will versus compulsion (Heather and Segal, 2016; Heather, 2017; Levy, 2013; Sripada, 2018; Pickard et al., 2015; Pickard, 2017), Flanagan echoes Weinberg in describing the first-person experience of addiction as involving normative failure (Flanagan, 2013). Firstly, he suggests that addicts feel shame because of a failure of agency: they decide not to use, and yet use. Secondly, they fail to live up to the ideals they have for a good life, for instance by letting others down through some action taken to maintain their addiction: what Flanagan calls a failing of moral quality. This latter part of Flanagan's model restates the lay view of addiction as interfering with role obligations such as holding down a job or caring for a family (Quintero and Nichter, 1996 p. 221) which is also one of the US Diagnostic & Statistical Manual of Mental Disorders (DSM-5) criteria for addiction (Fraser et al., 2014 p. 41, American Psychiatric Association, 2013). It chimes, moreover, with a material culture approach emphasising the role of substances in making and maintaining social relationships (Warde, 2015, Douglas, 2003 (1966), Miller, 2002). Both Weinberg and Flanagan are concerned with alcohol, cocaine and opiates, but smoking has been increasingly medicalised as an addiction (Bayer and Colgrove, 2004, p. 36) and I will argue that Flanagan's model of addiction experienced as normative failure also has explanatory power in relation to nicotine use.

## 2 Methods

My overall approach was naturalistic enquiry and specifically ethnography, the key feature of which is lengthy and repeated engagement with field sites and research participants (Messac et al., 2013 p. 184). My research took place in an economically depressed small town in a rural, formerly coal-mining area of the North East of England and a contrasting second site: a deprived urban neighbourhood three miles from the centre of a North West city. Increasing spatial segregation by income (Fahmy et al., 2011; Quillian, 2012) means that the social gradient in smoking is reflected in place-based inequalities (Glenn et al., 2017; Pearce et al., 2012; Thompson et al., 2007) so that by selecting areas of high deprivation for this study, I was also choosing areas with high rates of smoking. I made fifty visits during 2017 and 2018 to venues including community centres, shops, libraries and working men's clubs; however most of my time was spent at a community project in each field site serving mainly unemployed people and over half the interviewees were service users and staff from these two venues (n = 36/59). Ethical approval for the research was obtained from the Economics, Law, Management, Politics and Sociology (ELMPS) Ethics Committee at the University of York. I talked research participants through an information sheet which explained the purpose of my study, the identity of the funders, anticipated use of data and other issues before obtaining written consent (ASA, 2011). Research locations have been obscured and names and other details of participants changed in the text to protect anonymity.

As well as making notes of conversations and observations during each visit, I recorded interviews with smokers and recent quitters about their lives and particularly their smoking and e-cigarette histories; the quotes reproduced in this article are taken from these interviews (n = 59). My goal was to create a dialogic relationship (Riessman, 2008 pp. 23–26) and wherever possible I had regular conversations with research participants over a period of eighteen months. The sample was a convenience one and included 26 women and 33 men; the age range was 18–77 with a mean of 40; all research participants were white, including two non-UK nationals. Thirty-five participants were interviewed in the North West and 24 in the North East. At the time of first interview, 29 were unemployed, 18 were employed in routine and manual work and 12 were employed in administrative roles. Twenty-three smokers had tried an e-cigarette but discontinued use, 19 had switched to e-cigarettes, 14 had never tried one and three were dual users of tobacco and e-cigarettes. These relative numbers should not be seen as representing local e-cigarette take-up since I disproportionately recruited people with e-cigarette experience. I also interviewed staff from 20 e-cigarette shops or market stalls which sold e-cigarettes (n = 24) and observed customer interactions.

The tobacco landscape included both legal tobacco sold in a wide range of outlets (newsagents, convenience stores, supermarkets and fuel stations) and illicit tobacco smuggled into the UK after either being sold legally in countries with lower tax regimes or manufactured for the criminal market. E-cigarettes were sold in the same outlets as tobacco with the addition of street markets and specialist vape shops. Research participants used and discussed either second-generation, refillable, pen-shaped e-cigarettes such as the Ego CE4, costing from £5 ($6) upwards, or third-generation models for £20-plus, which have a larger battery, offer variable power to customise the vaping experience and can produce greater amounts of vapour and more effective nicotine delivery (Wagener et al., 2017). The ‘cigalike’ and ‘pod’ were rarely mentioned and are not discussed here. It was possible to buy 10 ml of e-liquid for £1 which made vaping potentially much cheaper than smoking, and research participants were generally aware of this price advantage.

Ethnographic analysis proceeds not so much by thematic analysis as by taxonomies and classifications, looking at what people have in common and how they differ (Prentice, 2010); interview data are analysed in the context of other data collected in informal conversations, through reading local histories and newspapers etc. My analysis involved close reading of narratives and comparing individual histories of smoking and vaping, taking other factors into account such as age, gender, class position (e.g. blue/white collar) and employment as well as local culture and history in order to understand the interplay of different variables and situate smoking and vaping within broader value systems, or what Kleinman terms local moral worlds (Kleinman, 2010 p. 375). I treated individual histories analytically as units rather than fragmenting them into thematic categories (Mishler, 1996; Riessman, 2008 p. 12) before generalising to theoretical perspectives (Melia, 2010).

## 3 Findings

### 3.1 Addiction as a moral problem

Many smokers were deterred from e-cigarette use by the fact that the vapers they knew continued to use e-cigarettes indefinitely: trainee Teaching Assistant Nicola (40s) was unwilling to switch from smoking to vaping: ‘*because I know people that have give* [sic] *up, and they still need e-cigs for eight years, so they're supposed to be like, gradually get you down, but people are getting addicted to them.’* Similarly, Patrick (18), who had come into the community project to look for work, told me his own e-cigarette hadn't helped him give up smoking because *‘you kind of pick that up more than you would have a normal cigarette’.* Patrick was one of many people who judged addiction according to the frequency and length of time spent vaping compared to prior smoking (see for instance Lucherini et al., 2017 p. 85, Rooke et al., 2016 p. e63). Patrick conceded that some of his friends and family had stopped smoking using an e-cigarette but he expressed concern that *‘whereas they've become not addicted to cigarettes, they've kind of become addicted to the shisha-pen, like, the e-cigarette’.* Administrative worker Oliver (28) had briefly switched to e-cigarettes but he too equated smoking and vaping: *‘I just basically replaced it really … I was still smoking that as much as I was smoking, if not more to be honest, because you could smoke inside as well with the e-cig … I thought if I'm going to stop, I need to actually stop.’* Housing support worker Lisa (30s) rejected the idea of using tobacco substitutes; she had tried an e-cigarette but returned to smoking when it broke. She told me: *‘If I went onto the e-cig I think I would probably use it the same amount of time as I would a cigarette … I think if you're going to be a smoker, you're a smoker, if you're not, you're not, you don't need an alternative* *…. You give up, you give up, that's that’.*

Another indication that addiction was seen as a problem was the fact that most vapers were planning to reduce the nicotine content of their e-liquid (Soar et al., 2019) and/or to give up the e-cigarette completely. Many told me proudly that they were using a liquid with a lower nicotine concentration than when they first started. Nathan (20s) described his decision to upgrade to a better e-cigarette as follows: *‘I wasn't getting enough vapour, was the main thing, but also wanted to drop down the nicotine as well, because I started on sixteen* [mg of nicotine per ml of liquid] *and now I'm down to a three’.* Kitchen worker Hannah (24) stopped smoking using an e-cigarette shortly before I first met her; she was keen to get her nicotine down, and when I spoke to her a year later, she had achieved this but she now kept her e-cigarette in her tabard pocket and used it continuously rather than once an hour; the fact that she was using a lower percentage of nicotine was more important to her than the inconvenience of needing to vape more frequently.

Semi-retired skilled fitter Stephen (67) described his addiction to nicotine explicitly as a moral problem:

*‘I was using [e-cigs] and the cigarettes you see, until I decided one day, it was a Sunday morning, I was sitting outside having a cup of tea, and I was trying to roll a cigarette out of the dust of the pack, inside the packet, scraping the dust out of the inside of the packet and trying to roll one, which was the first one of the day, and I said Steve, why are you a slave to this? And I actually spoke to myself…. And I said look, I'll see if I can last ‘til dinner time with the electronic cigarette, and I lasted till dinner time, and then I just carried it on from there, and it's been over two year … but now I'm addicted to this! [laughs] … I feel a slave to this now, which I was a slave to the tobacco before, I've changed one habit to another … I'm looking forward to the future when I can actually give this up … when I've decided I'll give it up, I will give this up, just so long as I do not go back to the old’.*

Stephen articulated most clearly the moral problem of addiction as a failure of the will; however there were still echoes in his narrative of the belief, often linked to a masculine narrative of mastery, that he could ‘just stop’ (White and Baird, 2013 p. 757, Thirlway, 2016 p. 110). As Keane points out, no-one is an addict until they want to stop and cannot (Keane, 2006 p. 107); this is probably why young smokers associate addiction negatively with older smokers and see themselves as in control and able to quit (Rugkasa et al., 2001; Amos et al., 2006; Scheffels and Schou, 2007). Bar worker Graham (23) had cut down to three cigarettes a day: *‘I never really crave them, I don't feel, oh I need a cig, I never feel like that’,* he said*.* However when he tried to quit he soon relapsed, and when I saw him again a few months after our first interview, his smoking had increased considerably. Office worker Hayley (24) only smoked when she went out at weekends and occasionally during the day if work was stressful. She told me: *‘I think in a way I sort of deny the fact that I do like to smoke. If I go to the doctor's or anything and they say are you a smoker, I'll say no … Yeah, because I class a smoker as someone that addicted, and I'm not addicted … So I do class as a smoker it would be like something you do all the time. And that's why I class myself as not.’*

## 4 Keeping costs down

The shopping centre in the north-west site offered tobacco and e-cigarettes for sale at a range of prices, right down to the £1 liquids in discount stores and the illicit tobacco sold on the street corner. Research participants' tobacco buying strategies included economy cigarettes, roll-your-own (RYO) and illicit tobacco, reflecting transnational trends (Gilmore et al., 2015; Young et al., 2012). They all smoked economy brands, like bookkeeper Jackie (53), who smoked [Brand A] *‘because they were cheaper, and they didn't make us cough as much as the other [cheap ones]’.* If they or their relatives could afford to go abroad, they brought home suitcases full of cheap tobacco. Employment adviser Tracey (50) told me: *‘It's always been [Brand B], but then they got incredibly expensive, so I'm on [Brand C] now.*’ She took a pack out of her bag: ‘*[Brand B], only because they're duty free … they're about a tenner a pack I think now, so yeah. Mum came back from Benidorm yesterday … she's bought some for herself … she brought* [partner] *four hundred, she bought me two hundred. It's ridiculously expensive, but this is why I want to stop again.’* Twenty-three out of fifty-nine research participants bought illicit tobacco: 20 cigarettes for £3.50 ($4.60) instead of £8-£12, or 50 g of illicit rolling tobacco i.e. enough for three to ten days for £9 ($12) rather than £20. Smokers made their cigarettes go as far as possible; unemployed chef Laura (30s) told me: ‘*I went into the shop yesterday and asked could I have the cheapest brand of cigarettes … my mam's on holiday* [visiting me], *so money's a little bit tight … so just, I spare my cigarettes out at the minute.’* People ‘nipped’ (North East), ‘pecked out’ or ‘dimped’ (North West) cigarettes i.e. extinguished them half-way through by gently screwing the lighted end against a wall or other hard surface and saving the second half for later, or kept their intake down by rolling a day's supply of cigarettes every morning and keeping it in a small tin in their pocket or bag.

Research participants spoke of the benefits of e-cigarettes mainly in terms of money savings, often in relation to family responsibilities. At a North West adult education centre, I chatted during their break to four women training to be teaching assistants, three of whom smoked whilst the fourth, Natalie (34) had switched to an e-cigarette four years previously: *‘I was just sick of wasting the amount of money you waste on cigarettes. Especially if you smoke ten, fifteen a day, over the weeks, if you've got two kids … ’* Another mother of two, support worker Rachel (30) was only an occasional smoker but was thinking of switching to e-cigarettes: *‘Because recently I've been smoking more, and I have bought them a couple of times as well, and they're really expensive, and I've really not got a lot of money - I think an e-cig would be brilliant for me’.* Another young mother, Rachel's friend Katie (27) who was out of work and receiving sickness benefits because of mental health issues, added: *‘I do want to try one of them electronic cigarettes, because, you know, I don't know how safe they are for you, but the money-wise … My friend, she was smoking like say forty a day, she's been on one of them electric cigarettes for three months now and not had a cig. She said one liquid lasts her three weeks … when you think of how much she would have spent on cigs!’*

Whereas smokers with higher social status refer to concerns about future health, poorer smokers tend to speak of cost and current health problems as triggers to stop smoking (Vangeli and West, 2008). Of the nineteen people I interviewed who had switched to vaping, only two gave health as the reason: bookkeeper Jackie (53), who switched to vaping after she had tests for throat cancer (which were negative), and unemployed Gavin (19), who quit smoking after two years as a dual user because: *‘I could feel it when I was going for a run, me breathing was getting a bit lower’*. One reason why research participants did not extol the health benefits of switching may have been that many had chronic problems which would not be resolved by smoking cessation: barmaid Julie (57), who suffered from chronic obstructive pulmonary disease (COPD), first came across electronic cigarettes when her son (21) told her he had bought one to stop smoking. She hadn't known he was a smoker: *‘I burst into tears and told him he's ruining his life, blah de blah, you don't want to be like me, one of them old fashioned things'*. She told me about the savings she had made since switching and I asked if her COPD symptoms had improved: *‘It's not got any worse’*, she said tersely. Stephen, who had spent the past thirty years fitting PVC doors and windows, no longer needed antibiotics every winter for chest infections but had symptoms of what may have been COPD or silicosis linked to occupational exposures: *‘I still get breathless but I think it's something to do with the atmosphere that I work in, it's stone dust, plaster dust from old buildings'.*

Amongst those people who stopped smoking and switched to e-cigarettes, it was not enough to save money by vaping; they also had to vape as cheaply as possible. With regard to hardware, some successful switchers were still using a cheap pen-type e-cigarette three or four years after they quit smoking. Stephen, who had not smoked for two years, said the larger mod-types: ‘*don't seem to last long, them, they seem to break. There's too much goes wrong with them.’* He preferred to go through twelve to fifteen of the cheap ones a year, although he had found that the very cheapest were best avoided: ‘*You're better off paying £8.99* [$12] *than a fiver, there's a difference, it's stronger, it lasts, seems to be lasting longer’.* Claire, a cleaner in her fifties, was also happy with a basic second-generation e-cigarette: *‘About two year* [sic] *ago, I was in the shop and I seen* [sic] *one and thought right, I'm going to try one of them. I've had about fifteen of these –* [quit smoking] *two years in November’.* Unemployed Nathan (20s) went through sixteen of *‘them rubbish little* pens’ before he bought his *‘first proper one’* from a vape shop. Natalie bought a pen-type e-cigarette and continued to use the same model for four years; she first acquired a bigger one only a few months before we spoke: *‘The batteries didn't last very long on the small pen ones so I wanted something that would last longer, because when I was going on holiday I didn't want to be messing about’.* If research participants did buy an expensive model, it was a one-off purchase; Laura had bought a £70 e-cigarette but returned to smoking after it fell out of her pocket and broke; now she ‘spared out’ her cigarettes so she could spoil her mum. Julie was marched into a vape shop by her son to buy her first e-cigarette, but for the four years since, she had acquired further (third-generation) e-cigarettes only at Christmas and birthdays.

But it was in relation to e-cigarette liquids that people were most sensitive to price; retailers talked about customers buying a sophisticated e-cigarette but using a liquid more suitable for a basic pen-type (i.e. with a lower ratio of vegetable glycerine to propylene glycol) because they could buy this more cheaply. The thin liquid then caused the e-cigarette to spit and splutter. There appeared to be no direct link between poverty and the drive to save money: unemployed people living on £73 ($95) a week were least likely to switch to e-cigarettes; and it was often vapers in comparatively secure financial circumstances who spent time and energy on finding the cheapest supplies. Julie bought her starter kit from a vape shop but found a much cheaper liquid on the market in a seaside town fifty miles away, *‘so now I bob up there once every few months and get it in bulk’.* Retired pub manager David (68), whom I met in a Working Men's Club, told me his first e-cigarette was ‘*given me by a friend - he tried it and didn't like it, so he gave it to me. Then I got one off the lad that were sitting there* [points to recently vacated seat]’. He bought the liquids *‘from the shop across the way* [discount shop] *- any one, 1.99 - I've found a cheaper one now.*’ He fished several bottles out of his pocket and put them on the table. I asked him what flavour he liked: *‘Cherry – well if it's cheap enough I'll buy it, American blend, English blend … that's the tobacco one, the market stall were selling them cheap, five for a pound’.* His friend John (77), a retired mining manager, told me he took up the e-cigarette because: *‘It was cheaper, a lot cheaper … I go across the road, but now I'm going up to* [discount shop], *I hear they're only a pound a bottle’.* John's friends were also keen to tell me that he had tried vaping cooking oil on one occasion, which was not a success. Some local vape shop owners saw this emphasis on price rather than health as part of the economic north-south divide. One told me:

*‘Health issues here isn't a big thing, things are based on price whereas if you were to travel to the South East, they would say it's all health. So they're shouting health, health, health, whereas we're shouting price, price, price. Down there they will be “this is a much healthier option than smoking” whereas here it's “they're cheaper than smoking”.’*

Another shop owner added*: ‘‘In the North East I think they're looking more for a bargain. The north is a bit more deprived; I think there is more money spent in the south.’* This is consistent with findings that smokers spend less on tobacco and vapers spend less on e-cigarettes in the North of England, even once socio-economic differences are accounted for (Kuipers et al., 2019; Jackson et al., 2019).

## 5 Refusing pleasure

Pleasure in the context of addiction is not a straightforward concept; Kennett et al. suggest that it plays a significant role but that this decreases over time as users become increasingly resentful or despairing of the effects of their substance use on their capacity to realize other values (Kennett et al., 2013 p. 10). As regards combusted tobacco, the way research participants talked about smoking suggested that pleasure was attached specifically to smoking with friends, usually whilst drinking. Some younger smokers limited their smoking to those occasions, but daily smokers like long-term unemployed friends Graham (58) and Paul (63) also identified smoking pleasure as linked to sociability. Paul told me: *‘When you go in the pub you want to smoke; you have a pint, you have a smoke’*. Graham added that he had people come over to his flat to ‘*drink and smoke and that sort of thing’.* Smoking to get a break or to address withdrawal symptoms was separate from smoking for pleasure: support worker Rachel (30) had a cigarette when she was *‘out having a drink’*, but also at work, where: *‘we'll have a little moan to each other, and the day just seems a bit better when we come back in’*. Some people made the distinction between functional/pleasurable smoking by using the cheapest available tobacco during the day but splashing out on a pack of their favourite ‘straights’ (ready-made cigarettes) for evenings out (also see Thirlway, 2016 p. 110). In fact three research participants adapted e-cigarettes to this system, using them as a cheap fix for their addiction and reserving cigarettes for special occasions. Two of these smoked when they had the money for tobacco and vaped when they ran out; a third, Jamie (30s) saved his cigarettes for the evening: *‘I can't afford to smoke so I'm going to have to get used to the e-cig … and it's more just really the saving money, because I can't afford to buy tabs* [cigarettes] *all the time … what I tend to do is try and smoke my e-cig during the day and then I'll have either a tab* [cigarette] *or rollie on a night.’*

Typologies of e-cigarette users have identified a sub-group who reject ostentatious equipment, large clouds and exotic flavours as part of a broader rejection of vaping as pleasure (Farrimond, 2017; Tokle and Pedersen, 2019; Thirlway, 2016 p. 109); almost all my research participants belonged to this group. Former smoker Ian (55) told me his e-cigarette was *‘just a little one, I don't need the big ones. I mean it really aggravates me when I see these people with big massive clouds of smoke coming out of their mouth, I just cannot see the point of that. I just smoke because me body tells us I want a hit and not because I want to make an impression’.* Dual user Jamie (30s) said: *‘I'm not interested in puffing out loads and loads of smoke … that's more of a novelty thing I think – as long as it gives us me fix’*. Gavin (19) unemployed, started vaping when he was fifteen to try to quit smoking and managed it aged seventeen: *‘I didn't want to get a big chunky one that filled out so much air, I'd rather have one that can help us stop smoking than I can show off with basically’*, he said. Gavin also complained about vapour clouds *‘in your face, blinding you’*; ostentatious vaping went against the norms of considerate smoking, itself a moral discourse (see for instance Lucherini et al., 2018 p. 1043, Poland, 2000). Employment adviser Tracey (50), who had managed to quit for three months using an e-cigarette but later relapsed to smoking, told me: *‘I won't have a fruity flavour, I just don't get the point, if I'm replacing a cigarette I want it to taste like a cigarette not have like strawberry-flavoured vapour wafting round, I don't get it’.* However, she went on to suggest she avoided sweet flavours because they might be more addictive: *‘I just think, because then you can become, if it's a pleasant flavour, you're going to want that more, so in my head I would be smoking that longer because that tastes nice’.* In a later conversation, Tracey also told me that she had stopped using her e-cigarette partly because it had started tasting bad; it turned out that she had never changed the coil, which has to be changed every few weeks. When I pointed out that this would explain the bad taste, she told me she thought it was better if it tasted bad, otherwise she would get addicted. In avoiding sweet flavours and accepting the unpleasant taste of a burnt-out coil, Tracey made it clear that she distrusted pleasure, reasoning that it would only worsen her addiction. Many users were keen to stress that they were only using an e-cigarette to medicate their addiction; in this context, sweet flavours were seen as frivolous as well as potentially addictive. E-cigarette retailer Emma (30s) told me *‘old ladies’* bought tobacco-flavoured e-liquids rather than the fruity or sweet flavours *‘because they don't do it for pleasure, they do it as a smoking substitute’.*

## 6 Discussion

Concern about addiction as ‘part of a broader discomfort about not being dependent and losing control’ (Rooke et al., 2016p. e63) runs through first-person accounts of nicotine use whether delivered through combusted tobacco, e-cigarettes or nicotine replacement therapy (Yerger et al., 2008; Amos et al., 2006; Lucherini et al., 2017; White and Baird, 2013; Smith et al., 2015; Hanninen and Koski-Jannes, 1999). But is the failure of agency linked to addiction necessarily experienced as morally problematic? Flanagan sees it as leading automatically to a sense of personal failure and therefore shame; sub-Saharan African philosophy's account of addiction offending against the life-force (a concept akin to energy, industriousness or self-realisation) suggests this idea has cross-cultural validity beyond western ethicists' Kantian accounts of addiction degrading rationality or autonomy (Metz, 2018). Conversely, social scientists concerned with the social production of addiction theorise the shame of addiction as the internalisation of external stigma (Matthews et al., 2017) or make the Foucauldian argument that claims that addicts' freedom and autonomy are compromised conceal the reality that state institutions disapprove of their choices (Keane, 2002; Reith, 2004; Rooney, 2003; Fraser et al., 2014). But whether shame is inherent to addiction or socially imposed, the loss of control implied by addiction is experienced as a moral problem by many smokers and is not necessarily resolved by switching to another form of nicotine use.

The other reason why addiction was a moral problem concerns Flanagan's further argument that the shame of addiction relates to letting others down, or what I will call a failure of care. DSM-5 takes the view that tobacco use ‘rarely results in failure to fulfil major role obligations’ (American Psychiatric Association, 2013) but the literature reveals many instances of smoker guilt over a failure to be a good role model (Mcdermott et al., 2006 p. 435), to stay healthy for the sake of family (Thirlway, 2016 p. 110), to quit smoking in pregnancy (Coxhead and Rhodes, 2006 pp. 111–114, Irwin et al., 2005; Bottorff et al., 2013) or as I argue here, to exercise thrift as care i.e. to avoid the financial cost of addiction as a diversion of household resources (see also Robinson and Holdsworth, 2013 p. 49 on cost rather than health as motivation to quit). Social scientists take the view that everyday practices of consumption have complex social meanings (Zelizer, 1997; Wherry, 2008; Parry and Bloch, 1989; Maurer, 2006). Daniel Miller sees domestic provisioning as an expression of kinship, with thrift as the central expression of this relationship (Miller, 1998); I argue that the medicalisation of smoking as addiction (Bayer and Colgrove, 2004) has resulted in tobacco undergoing a category change from an unremarkable staple of working-class budgets to a morally suspect substance on which expenditure should either be minimised or justified as an occasional treat (or, as some research participants said, their ‘only pleasure’ - see also Bancroft et al., 2003 p. 1264, Stead et al., 2001 p. 340, Lang et al., 2007 p. 519). Research participants extended the same complex moral calculations to e-cigarettes, so that while the occasional purchase of an expensive e-cigarette was permissible, everyday spending on e-liquid refills had to be minimised.

There have been suggestions that more attention should be paid to sensorial pleasure in the study of addiction (Bunton and Coveney, 2011) and that it is a key aspect of e-cigarette use (Cox and Jakes, 2017; Bell, 2013). With regard to smoking, I found that the morally suspect status of tobacco as an addictive substance limited pleasure to social situations where hedonism is appropriate. Many smokers also viewed e-cigarette pleasure with suspicion because hedonism is symbolically linked with addiction, making it necessary for e-cigarettes to be conceptualised as medication in order to be morally acceptable. As regards non-sensorial pleasure (Coveney and Bunton, 2003), two forms of moral satisfaction are relevant to e-cigarette use. The first is the moral satisfaction of thrift and its capacity to alleviate concern about addiction as role performance failure by minimising the diversion of financial resources from the household. The second is the moral satisfaction of enjoying a less harmful alternative to smoking (Farrimond, 2017 p. 86, Tokle and Pedersen, 2019).

This brings me to a key part of my argument. Rather than presenting addiction shame as a universal condition (Flanagan, 2013), I suggest that concern about addiction is more likely to be a barrier to working-class than to middle-class smokers. The first reason is that for the middle-class, moral concern about addiction is offset by a strong imperative to pursue individual health and avoid combusted tobacco as part of efforts to distinguish itself from the working-class (Bourdieu, 1984; Thirlway, 2018). The benefit to health of replacing combusted tobacco with e-cigarettes therefore has inherent moral dividends in line with the dominant middle-class narrative of individualised health as moral virtue or ‘super-value’ (Lupton, 1995; Conrad, 1994; Petersen and Lupton, 1996; Crawford, 2006). For those middle-class smokers who switch to e-cigarettes, the fact that they are no longer smoking provides a moral justification strong enough to outweigh addiction shame. In addition the moral shame of spending on addiction and thereby diverting resources from the household may be greater in a less individualised working-class culture where caring for others is key to responsibility and respectability (Skeggs, 1997 pp. 163–164, Holdsworth, 2009 pp. 1858–9).

Other studies have identified typologies of vapers without linking these to specific social groups (Farrimond, 2017; Tokle and Pedersen, 2019), but ethnography requires interpretative depth (Panter-Brick and Eggerman, 2018 p. 234) rather than a paraphrase of what people say (Fassin, 2013 p. 122) and I suggest that the moralization of smoking (Rozin and Singh, 1999; Gostin, 1997) has had differential effects by class: for the middle class, smoking has been moralised primarily as a failure to pursue health, whereas for working class people who are less likely to feel they can control their health (Balshem, 1993, Chamberlain and O'neill, 1998), smoking has been moralised primarily in the extent to which it is seen as addiction. Peter Miller has argued that harm reduction is a middle-class paradigm promoting a duty to be healthy (Miller, 2001; Keane, 2003), but I see this as a practical as much as an ethical problem: the harm reduction approach overlooks the fact that those most affected by tobacco harm are more concerned about addiction than health.

Does this mean working-class smokers are unlikely to embrace e-cigarette use? Not necessarily, but if they do, it may be as much because of the price differential as the health advantages; somewhat paradoxically, they may also respond better to a medical model of e-cigarette use on the basis that it provides a moral justification for the use of an addictive substance, rather than to a model which emphasises pleasure (Farrimond, 2017). Higher tobacco prices are associated with greater take-up of electronic cigarettes (Liber et al., 2017; Stoklosa et al., 2016); refillable electronic cigarettes are currently cheaper than combustible cigarettes in the UK, but globally this is not the case. Whilst concern has been expressed about the initial cost of a second-generation e-cigarette as a barrier to use (Liber et al., 2017 p. 160, Thirlway et al., 2019; Dawkins et al., 2019), my findings suggest that ongoing, everyday costs i.e. e-liquid refill affordability may be even more important. Ensuring through differential tax regimes and regulation that vaping is significantly cheaper than smoking may be key to addressing health inequalities, not simply because disadvantaged smokers lack financial resources but because minimising spend on their addiction may be a greater moral concern for them than minimising risk to their health.

## 7 Limitations

This study has discussed barriers to e-cigarette use only in relation to class; differences in the moral experience of addiction by gender, age and life stage have been touched upon but could not be fully explored here. As suggested by some vape shop owners' allusion to Northern exceptionalism, it may also be the case that the particular values which emerged in this study such as ‘thrift as care’ are more typical of the Northern England context than of working-class experience elsewhere (Kuipers et al., 2019; Jackson et al., 2019); however, these are also some of the highest-smoking areas and as such are a public health priority for the UK. It may also be the case that these values are relevant to other dominated groups and find an echo in very different international contexts of smoking and vaping (see for instance Yerger et al., 2008).
